# Supplementary figures and images for: ATP binding cassette transporters and uridine diphosphate glycosyltransferases are ancient protein families that evolved roles in herbicide resistance through exaptation
Source: PLoS One. 2023 Sep 21;18(9):e0287356. doi: 10.1371/journal.pone.0287356 (PMC10513242; doi:10.1371/journal.pone.0287356)

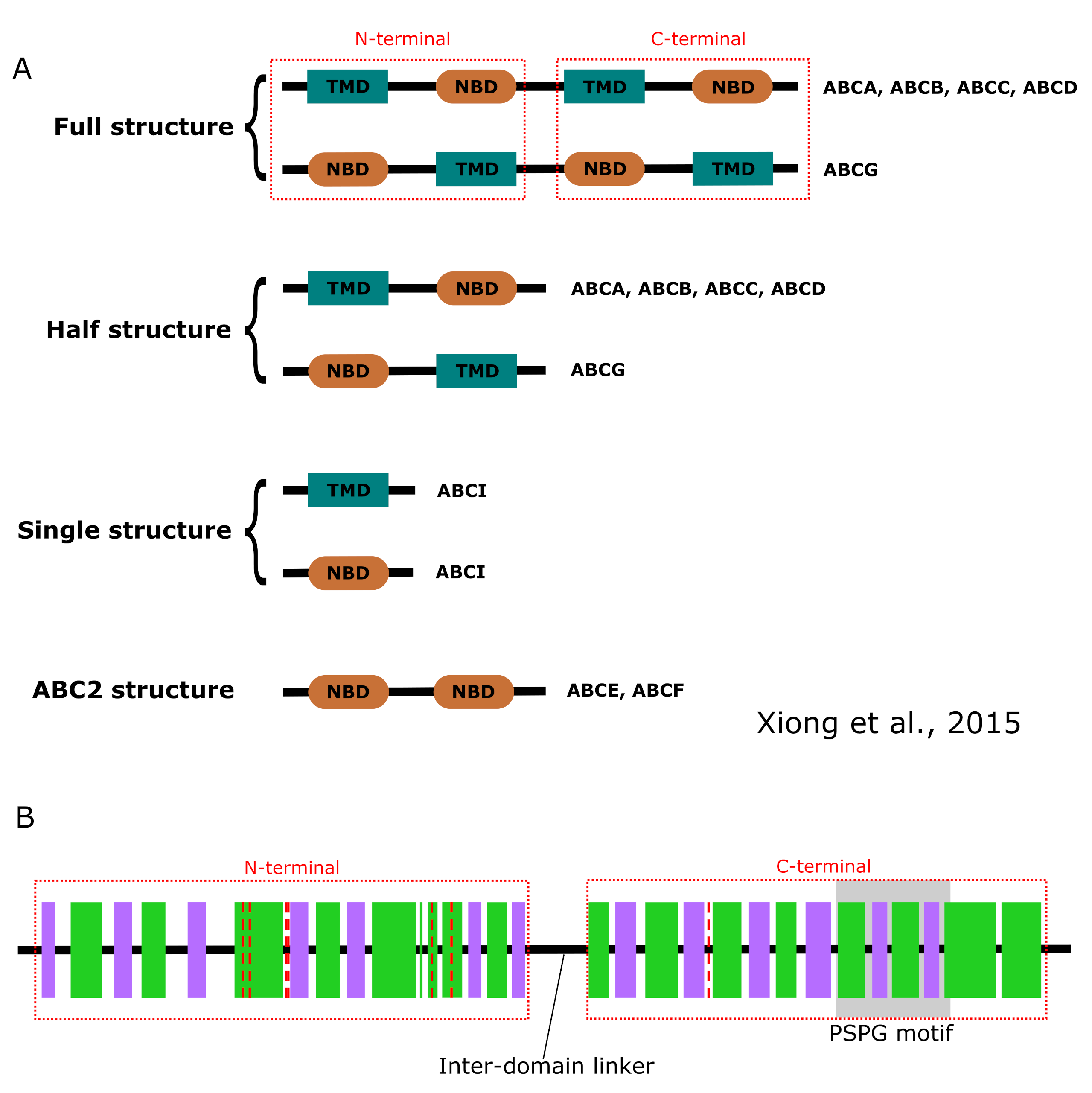

Supplement: S1 Fig — Diagrams of the typical ABC transporter (A) and UDP-glycosyltransferases (B). A shows the domain architecture of ABC transporters from each of the major sub-families in plants. The diagram is adapted from https://doi.org/10.1038/srep16724. B shows α-helices (green), β-strands (purple) and residues reported to form part of the acceptor pocket (red dashed lines). The PSPG motif reported to interact with the UDP-sugar donor is highlighted in grey. The structure is based on that of AtUGT72B1 reported in https://doi.org/10.1016/j.phytochem.2008.12.009. (TIF) [file pone.0287356.s001.tif]

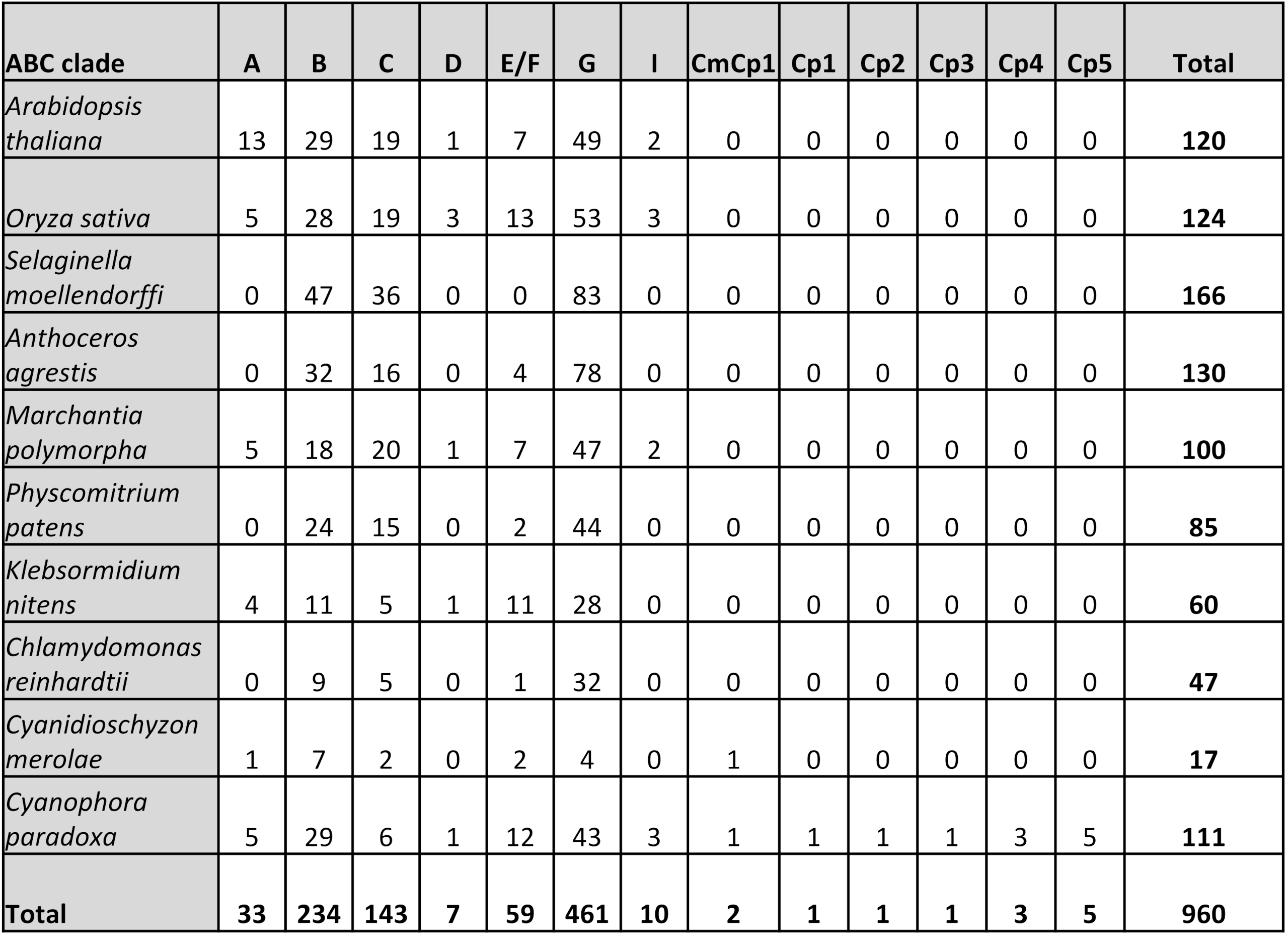

Supplement: S2 Fig — (TIF) [file pone.0287356.s002.tif]

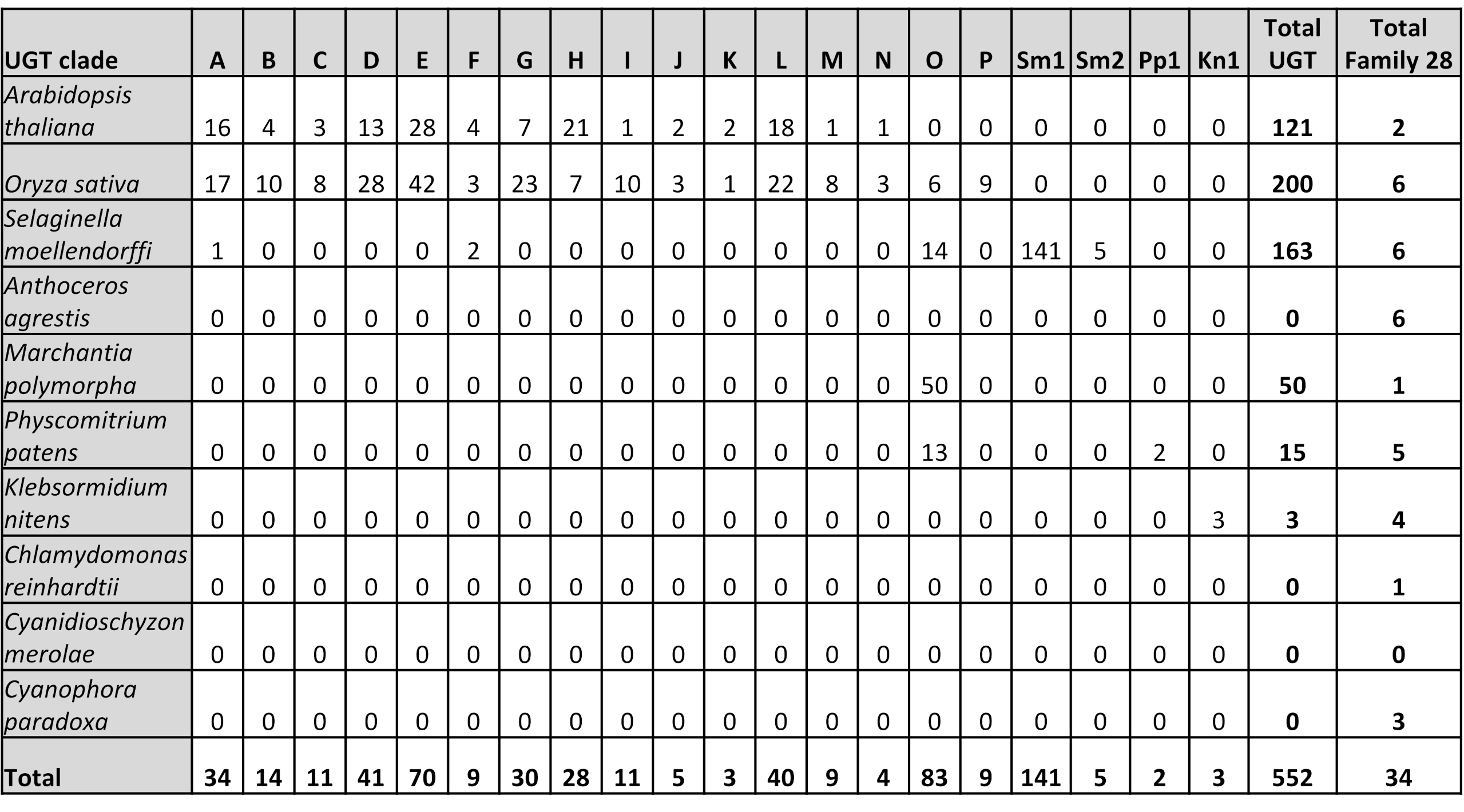

Supplement: S3 Fig — Number of Family 28 glycosyltransferases identified in each species is also given. (TIF) [file pone.0287356.s003.tif]
